# Expression of concern: Microchip-based structure determination of low-molecular weight proteins using cryo-electron microscopy

**DOI:** 10.1039/d4nr90037e

**Published:** 2024-02-14

**Authors:** Michael A. Casasanta, G. M. Jonaid, Liam Kaylor, William Y. Luqiu, Maria J. Solares, Mariah L. Schroen, William J. Dearnaley, Jarad Wilson, Madeline J. Dukes, Deborah F. Kelly

**Affiliations:** a Department of Biomedical Engineering, Pennsylvania State University University Park PA 16802 USA Debkelly@psu.edu; b Materials Research Institute, Pennsylvania State University University Park PA 16802 USA; c Bioinformatics and Genomics Graduate Program, Huck Institutes of the Life Sciences, Pennsylvania State University University Park PA 16802 USA; d Molecular, Cellular, and Integrative Biosciences Graduate Program, Huck Institutes of the Life Sciences, Pennsylvania State University University Park PA 16802 USA; e Department of Electrical and Computer Engineering, Duke University Durham NC 27708 USA; f RayBiotech Life, Peachtree Corners GA 30092 USA; g Applications Science, Protochips, Inc. Morrisville NC 27560 USA

## Abstract

Expression of concern for ‘Microchip-based structure determination of low-molecular weight proteins using cryo-electron microscopy’ by Michael A. Casasanta *et al.*, *Nanoscale*, 2021, **13**, 7285–7293, https://doi.org/10.1039/D1NR00388G.

The Royal Society of Chemistry is publishing this expression of concern in order to alert readers that concerns have been raised regarding the accuracy of some of the single particle data for N protein specimens presented in this article. An investigation is underway, and an expression of concern will continue to be associated with the article until a final outcome is reached.

 

Signed: Heather Montgomery, Managing Editor, *Nanoscale*

Date: 25 January 2024

## Supplementary Material

